# SELF-CARE AND WELL-BEING INTERVENTIONS FOR FAMILY CAREGIVERS OF FRAIL OLDER ADULTS IN A RURAL CONTEXT

**DOI:** 10.1093/geroni/igae098.3382

**Published:** 2024-12-31

**Authors:** Siqi Yang, Nicolas Cuevas, Shatakshi Shelar, Yu Lu, Bhagyashree Katare, Nasreen Lalani

**Affiliations:** Purdue University, West Lafayette, Indiana, United States; Universidad Nacional de Colombia, Bogota, Bogota, Colombia; Purdue University, West Lafayette, Indiana, United States; Purdue University, West Lafayette, Indiana, United States; Purdue University, Purdue University, Indiana, United States; Purdue University, Purdue University, Indiana, United States

## Abstract

The rapid aging of the population and limited access to healthcare in rural communities pose significant challenges for caregivers caring for older family members suffering from serious chronic illnesses. The heavy burden of care and multiple responsibilities cause poor coping, self-care and wellbeing outcomes and therefore need interventions to promote their resilience and overall quality of life. Our systematic review aims to explore interventions available in the existing literature to promote the self-care and well-being of family caregivers of frail older adults residing in rural underserved areas. Arksey & O’Malley’s methodological framework of scoping studies was employed for the analysis. Databases such as PubMed and Scopus were used to search the articles. PRISMA and Covidence tools were followed to review and analyze the study. The CASP tool was used to examine the quality of the study and consensus was established among three research assistants and the advisor in the team. Our findings include 24 selected articles, with the majority of studies conducted in the U.S. (63%). Interventions were categorized into four groups as digital-web-based interventions (7), telehealth interventions (6), psychoeducational interventions (9), and other interventions (2). The most common outcomes observed across these interventions were increasing caregiver knowledge, self efficacy and reducing caregiver burden. Our review emphasizes the need for more research using community-engaged and culturally based approaches to promote the wellbeing and resilience of rural caregivers.

